# Antimicrobial Efficacy of Cinnamon Essential Oil against Avian Pathogenic *Escherichia coli* from Poultry

**DOI:** 10.3390/ani13162639

**Published:** 2023-08-16

**Authors:** Gaia Casalino, Francesca Rita Dinardo, Francesco D’Amico, Giancarlo Bozzo, Antonella Bove, Antonio Camarda, Roberto Lombardi, Michela Maria Dimuccio, Elena Circella

**Affiliations:** Department of Veterinary Medicine, University of Bari “Aldo Moro”, S. P. Casamassima km 3, 70010 Valenzano, Italy; gaia.casalino@uniba.it (G.C.); francesca.dinardo20@gmail.com (F.R.D.); francesco.damico@uniba.it (F.D.); giancarlo.bozzo@uniba.it (G.B.); antonella.bove@uniba.it (A.B.); antonio.camarda@uniba.it (A.C.); roberto.lombardi@uniba.it (R.L.); michela.dimuccio@uniba.it (M.M.D.)

**Keywords:** colibacillosis, poultry, *Escherichia coli*, cinnamon essential oil, antimicrobial efficacy

## Abstract

**Simple Summary:**

The aim of this study was to evaluate the antimicrobial efficacy of cinnamon essential oil (CEO) against *Escherichia coli* (*E. coli*) strains isolated from poultry with colibacillosis. One hundred and seventeen strains isolated from laying hens, broilers, and turkeys and belonging to serogroups O78, O2, O128, O139, which are often responsible for avian colibacillosis, were analyzed. The minimum inhibitory concentration (MIC)_50_ and MIC_90_ of CEO were evaluated by testing each bacterial strain at cell densities of 10^8^ CFU/mL and 10^6^ CFU/mL, respectively. At 10^8^ CFU/mL, MIC_50_ and MIC_90_ were, respectively, 0.4 and 0.5 µL/mL for the strains from laying hens, and 0.5 and 0.6 µL/mL for strains from turkeys. MIC_50_ and MIC_90_ corresponded to 0.5 µL/mL for the strains isolated from broilers. Grouping the strains according to the serogroup, MIC_50_ and MIC_90_ were 0.4 and 0.5 µL/mL for strains belonging to serogroups O78, O2, and O128. A concentration of 0.5 µL/mL of CEO corresponded to both MIC_50_ and MIC_90_ for strains belonging to serogroup O139. MIC_50_ and MIC_90_ of CEO were 0.3 and 0.4 µL/mL respectively for strains tested at the cell density of 10^6^ CFU/mL, regardless of the bird species of origin. According to the serogroups, MIC_50_ and MIC_90_ were 0.3 and 0.4 µL/mL for strains belonging to serogroups O78 and O2. A concentration of 0.4 µL/mL of CEO corresponded both to MIC_50_ and MIC_90_ for strains belonging to serogroups O139 and O128. This study showed that CEO has effective antibacterial activity against pathogenic *E. coli* in poultry.

**Abstract:**

Colibacillosis, caused by *E. coli*, is responsible for economic losses in the poultry industry due to mortality, decreased production, and the cost of antibiotic treatments. Prevention of colibacillosis is based on improved biosecurity measures and the use of the vaccine performed with O78 *E. coli* strains, which is responsible for most cases of colibacillosis. Recently, there has been increased interest in other infection control methods, such as the use of natural compounds. The aim of this study was to evaluate the antimicrobial efficacy of cinnamon essential oil (CEO) against *E. coli* strains isolated from poultry. The MIC_50_ and MIC_90_ of CEO were determined by testing 117 strains belonging to serogroups O78, O2, O128, O139, isolated from laying hens (91 strains), broilers (10 strains), and turkeys (16 strains). The bacterial strains were tested at cell densities of 10^8^ and 10^6^ CFU/mL. At the cell density of 10^8^ CFU/mL, MIC_50_ and MIC_90_ were 0.4 and 0.5 µL/mL for most of the tested strains, while they corresponded to 0.5 µL/mL for all strains isolated from broilers and for strains belonging to serogroup O139. At the cell density of 10^6^ CFU/mL, MIC_50_ and MIC_90_ were 0.3 and 0.4 µL/mL, regardless of bird species of origin and for strains belonging to serogroups O78 and O2. In addition, a concentration of 0.04 µL/mL of CEO corresponded both to MIC_50_ and MIC_90_ for strains belonging to serogroups O139 and O128. Based on these results, cinnamon essential oil showed an effective antibacterial activity against *E. coli* strains from poultry and could find field application for the prevention of colibacillosis.

## 1. Introduction

*Escherichia coli* (*E. coli*) is a commensal microorganism that colonizes the lower gastrointestinal tract of mammals and birds shortly after birth and acts as a symbiont involved in the synthesis of vitamins needed by the hosts [1]. *E. coli* can also enrich its accessory genome with virulence genes (VG) that allow it to adapt to unfavorable environmental conditions by colonizing even extra-intestinal organ niches [2]. Certain serotypes, generally having different virulence factors [3,4] are more frequently associated to colibacillosis. Therefore, *E. coli* strains are classified into two main groups: intestinal pathogenic *E. coli* (InPEC) and pathogenic *E. coli* extra-intestinal (ExPEC) [5]. A subgroup of the latter, which is called avian pathogenic *E. coli* (APEC), is particularly relevant to the poultry industry because it causes colibacillosis, a disease with significant economic losses due to mortality and decreased productivity of the affected birds. The most frequent clinical forms of colibacillosis are as follows: yolk sac infection (omphalitis) in broiler chicks; septicemia in broiler chicks and adults; reproductive tract infections (salpingitis) in laying hens; respiratory infections, predominantly air sacculitis in both broilers and layers [5,6], and lesions in other visceral organs [6]. APEC strains use several virulence and pathogenesis factors, mainly adhesins, iron acquisition systems, and toxins [7]. These factors facilitate the evasion of host immune responses and systemic spread of APEC, enabling infection in chickens [7]. In addition to these factors, secretion systems (type III and VI), quorum sensing (QS) systems, transcriptional regulators, two-component systems, and metabolism-associated genes contribute to the APEC pathogenesis in chickens [8,9,10,11]. APEC virulence factors are also involved in mechanisms of resistance to antibiotics, such as β-lactams and colistin, which may pose a high risk to humans due to the transmission of antibiotic-resistant bacteria and genes through the food chain [12]. O1, O2, and O78 are the most common antigenic serotypes of APEC in poultry [13,14]. However, other serotypes are associated with colibacillosis in poultry, such as *E. coli* O111, which causes severe septicemia and polyserositis in hens [15], *E. coli* O128 and O139 isolated from broilers with a history of respiratory symptoms and pericarditis, peri-hepatitis, and air sacculitis [16,17]. A major predisposing factor for systemic APEC infections is stress, which can be induced by a variety of agents or by inappropriate husbandry practices [18]. Poor hygiene and lack of biosecurity measures in herd management and among farms may promote the spread of the infections and virulence determinants.

Candidate vaccines produced using pathogenic strains isolated from birds of affected flocks have been tested for prevention of colibacillosis [19,20,21,22]. Recently, a commercial live vaccine performed with O78 *E. coli* strain and registered in the EU in 2013 [23] was made available in most countries namely Italy, Germany, Spain, and France where laying hens are intensively reared. The vaccine provides effective protection in case of challenge with O78 wild strains [24]. However, it is less effective against infections due to strains belonging to other serogroups [25]. A study using the live commercial O78 vaccine and an inactivated candidate vaccine containing *E. coli* strains O18, O78, and O111 showed a better level of protection against colibacillosis conferred by combining the two types of vaccines rather than administering them separately [26]. Control of colibacillosis has historically been achieved using different classes of antimicrobials. However, considering the increase in antimicrobial resistance related to the excessive use of antibiotics, the potential risk of transmission of resistant bacteria to humans through the consumption of foods of animal origin, and the possible exposure of veterinarians and farmers to animals contaminated with antibiotic-resistant bacteria [27], alternative control strategies are recommended as part of a “One Health” approach that relies on integrated and unifying prevention measures to protect animal and public health [28,29]. In addition, economic losses occur in poultry farms due to compliance with the withdrawal period during and after the treatment.

Therefore, the poultry industry is currently focused on eliminating the use of antibiotics, thus seeking innovative management systems [30]. The production of antibiotic-free broiler has increased due to consumer perception that poultry meat is qualitatively superior to conventionally produced meat [31,32]. Alternative methods to antibiotics for the control of bacterial diseases in poultry farms could be natural substances such as some essential oils that, due to their potential antimicrobial properties, could find application in poultry phytotherapy [33]. Cinnamon has potential antibacterial activity that seems to be related to cinnamaldehyde, also known as cinnamic aldehyde, which is the main chemical constituent of cinnamon plants. The concentration of cinnamic aldehyde depends on the species of cinnamon and the part of the plant used to extract the essential oil [34,35,36]. Other bioactive compounds such as coumarins, alkaloids, tannins, and phenols that are constituents, although in smaller amounts than cinnamaldehyde, may contribute to the beneficial effects of cinnamon plants [37,38]. The aim of this study was to evaluate the antimicrobial efficacy of cinnamon essential oil (CEO) against APEC strains isolated from laying hens with colibacillosis.

## 2. Material and Methods

### 2.1. Bacterial Strains Used for the Experiment

One hundred and seventeen *E. coli* strains were used for the analysis. They were from the bacterial strain collection of the Avian Diseases section, Department of Veterinary Medicine, Valenzano, BA, Italy. All strains were isolated between 2001 and 2022 from laying hens, broilers, and turkey that died because of colibacillosis. Strains were grown on MacConkey agar (Oxoid, Basingstoke, UK) and incubated at 37 °C for 24 h. Each colony morphologically compatible with *E. coli* was selected and transferred onto nutrient agar (Oxoid) and incubated at 37 °C for 24 h. The colonies were tested for indole and gas production, and oxidase activity. Gas/indole-positive and oxidase-negative colonies were identified as *E. coli* using the API-20E biochemical gallery (bio Mérieux, Marcy l’Étoile, Lyon, France). Each strain was stored in Brucella broth (Oxoid) at −20 °C and glycerol (10%). The strains selected for the experiment belonged to some serogroups frequently responsible for colibacillosis in poultry. In detail, 54 strains belonged to serogroup O78, 37 to serogroup O2, 19 to serogroup O139, and 7 to serogroup O128.

### 2.2. Preparation of Bacterial Suspensions

Before analysis, all strains were grown on tryptic soy agar (TSA) (Oxoid, Basingstoke, UK) at 37 °C overnight. A bacterial suspension of 0.5 McFarland standard corresponding to 1–2 × 10^8^ CFU/mL [39] was prepared from each strain, using sterile saline solution (0.9%). Starting with a cell density of 10^8^ CFU/mL, bacterial suspensions of 10^6^ CFU/mL were obtained using stepwise dilutions.

### 2.3. Cinnamon Essential Oil and Preparation of Medium

Commercial (ERBA VITA GROUP S.p.A., Chiesanuova, San Marino) hydro-distilled pure cinnamon (*Cinnamomum zeylanicum Blume*) essential oil 100% pure (CEO) was used for the experiment.

The efficacy tests were performed on Muller–Hinton agar (Oxoid) reconstituted according to the manufacturer’s instructions and autoclaved at 121 °C for 15 min. The broth was heated to 50 °C in a thermostatic bath before adding CEO in different volumes according to the concentrations to be tested.

### 2.4. Preliminary Test

A preliminary efficacy test was performed using 4 strains of *E. coli* belonging to serogroups O2 (2 strains) and O78 (2 strains). Bacterial suspensions with cell density of 10^8^ CFU/mL and 10^6^ CFU/mL, respectively, were prepared from each strain.

According to a previous study [40], 10 µL of suspension with cell density of 10^8^ CFU/mL were spot inoculated on Muller–Hinton agar containing essential oil in concentrations from 0.01 to 1% (0.01, 0.05, 0.1, 0.5, 1%) corresponding to 0.1 µL/mL to 10 µL/mL (0.1, 0.5, 1, 5, 10 µL/mL), respectively.

Ten µL of 10^6^ CFU/mL bacterial suspension was spot inoculated on the oil-containing medium in concentrations ranging from 0.005 to 0.5% (0.005, 0.01, 0.05, 0.1, 0.5%) corresponding to 0.05 to 5 µL/mL (0.05, 0.1, 0.5, 1, 5 µL/mL).

Each suspension was inoculated simultaneously on oil-free Muller–Hinton agar, as positive control for bacterial growth. All plates were incubated at 37 °C for 24 h under aerobic conditions. The inhibitory activity of CEO was assessed by bacterial growth or nongrowth in the spot.

### 2.5. Preparation of the Efficacy Tests

**Figure 1:**
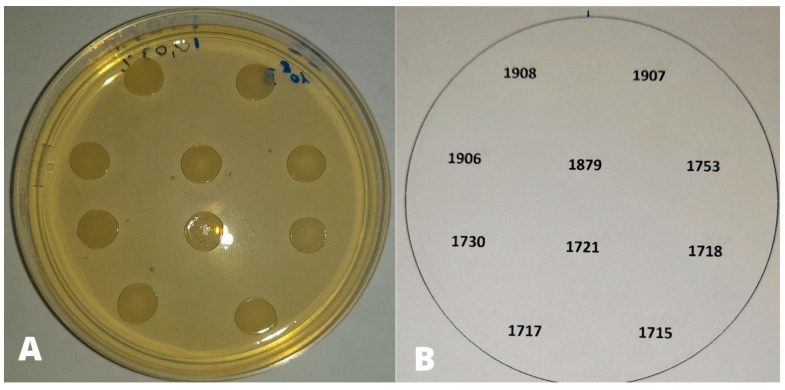
(**A**). Growth of strains in the corresponding spots; (**B**). Numbered grid for strain identification.

Based on the results obtained in the preliminary test, concentrations of CEO from 0.01 to 0.08% (0.01, 0.02, 0.03, 0.04, 0.05, 0.06, 0.07, 0.08%) corresponding to 0.1 to 0.8 µL/mL (0.1, 0.2, 0.3, 0.4, 0.5, 0.6, 0.7, 0.8 µL/mL) and 0.01 to 0.05% (0.01, 0.02, 0.03, 0.04, 0.05%) corresponding to 0.1 to 0.5 µL/mL (0.1, 0.2, 0.3, 0.4, 0.5 µL/mL) were chosen to assess the sensitivity of each strain at cell densities of 10^8^ CFU/mL and 10^6^ CFU/mL, respectively. The bacterial suspensions were spot inoculated on the medium. Each suspension was inoculated simultaneously on oil-free Muller–Hinton agar, as positive control for bacterial growth. After 24 h at 37 °C in aerobic conditions, the results were read by evaluating the efficacy of CEO based on strain growth/no growth of the strains in the spot. A numbered grid placed under the plate was used for both inoculation and strain identification (Figure 1). Each experiment was carried out twice on two different days.

### 2.6. Statistical Analysis

Inhibition data were analyzed by univariate statistical analysis (Pearson’s chi-square test and Fisher’s exact test for independence). Values of *p* < 0.05 were considered statistically significant. Statistical analyses were performed using SPSS 13 software for Windows (SPSS Inc., Chicago, IL, USA).

## 3. Results

### 3.1. Preliminary Tests

All strains tested at a cell density of 10^8^ CFU/mL were inhibited by essential oil at concentrations ranging from 10 to 1 µL/mL (10, 5, 1 µL/mL). Only one strain belonging to serotype O78 grew in the presence of 0.5 µL/mL essential oil. All strains grew with 0.1 µL/mL of essential oil.

At the cell density of 10^6^ CFU/mL, all strains were inhibited by CEO from 5 to 0.5 µL/mL, while they grew with concentrations of 0.1 and 0.05 µL/mL of CEO.

### 3.2. Efficacy Tests

**Figure 2:**
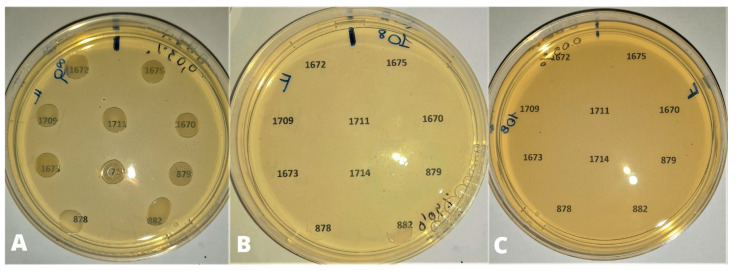
Bacterial density of 10^8^ UFC/mL. (**A**) 0.03% (0.3 µL/mL) of CEO: no inhibition of strain growth; (**B**) 0.04% (0.4 µL/mL) of CEO: inhibition of growth except for strains 878 and 882 (partial inhibition); (**C**) 0.05% (0.5 µL/mL) of CEO: inhibition of bacterial growth.

MIC_50_ and MIC_90_ were determined by testing different cell densities of *E. coli* strains. At 10^8^ CFU/mL, MIC_50_ and MIC_90_ of CEO were 0.4 and 0.5 µL/mL, respectively (Figure 2) (Table 1). 

Considering the whole strain pool, only four strains out of 117 strains (3.41% of the population) were inhibited at concentration of 0.3 µL/mL. All tested strains were inhibited at concentrations higher than 0.5 µL/mL. Grouping the bacterial strains according to the bird species of origin, MIC_50_ and MIC_90_ of CEO were, respectively, 0.4 and 0.5 µL/mL for strains from laying hens, and 0.5 and 0.6 µL/mL for strains from turkeys. MIC_50_ and MIC_90_ corresponded to 0.5 µL/mL for all strains isolated from broilers. 

Grouping the strains according to the serogroup, (Table 2), MIC_50_ and MIC_90_ were 0.4 and 0.5 µL/mL for strains belonging to serogroups O78, O2, and O128. One (2.7%) and three (5.56%) strains belonging respectively to serogroups O2 and O78 were inhibited by 0.3 µL/mL of CEO. Concentration of 0.5 µL/mL of CEO corresponded to both MIC_50_ and MIC_90_ for strains belonging to serogroup O139.

**Figure 3:**
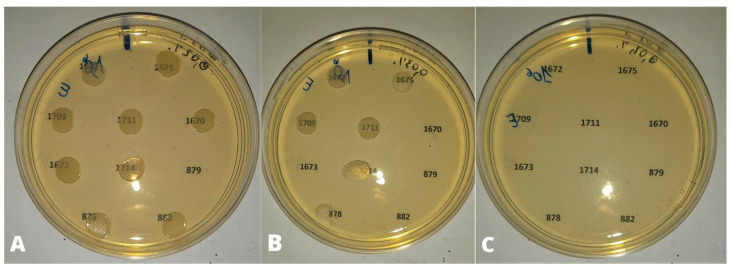
Bacterial density of 10^6^ UFC/mL. (**A**) 0.02% (0.2 µL/mL) of CEO: no inhibition of bacterial growth except for strain 879; (**B**) 0.03% (0.3 µL/mL) of CEO: growth inhibition of strains 1670, 1673, 879, 882; (**C**) 0.04% (0.4 µL/mL) of CEO: inhibition of bacterial growth.

MIC_50_ and MIC_90_ of CEO were 0.3 and 0.4 µL/mL respectively for bacterial strains tested at the cell density of 10^6^ CFU/mL (Figure 3) (Table 3), regardless of bird species of origin.

Eleven strains out of 117 (9.4%), including nine strains from laying hens, one from turkeys and one from broilers were inhibited by 0.2 µL/mL of CEO. All strains were inhibited by 0.4 µL/mL of CEO.

According to the serogroups (Table 4), the MIC_50_ and MIC_90_ were 0.3 and 0.4 µL/mL for strains belonging to serogroups O78 and O2. Seven strains out of 54 and four out of 37, belonging respectively to serogroups O78 and O2, were inhibited by 0.2 µL/mL of CEO (Table 4). Concentration of 0.4 µL/mL of CEO corresponded both to MIC_50_ and MIC_90_ for strains belonging to serogroups O139 and O128.

## 4. Discussion

Based on the data obtained, cinnamon essential oil (CEO) has effective antibacterial effects against pathogenic *E. coli* in poultry, regardless of the bacterial cell density used in the experiments. 

Concerning *Cinnamomum zeylanicum*, used in the experiments, the major components of the essential oil are cinnamaldehyde (88.2%), benzyl alcohol (8.0%), and eugenol (1.0%), which have synergistic or additive effects [41,42]. The combination of these components allows cinnamaldehyde to penetrate the phospholipid bilayer of bacterial cell walls more easily and bind more readily to proteins, preventing them from performing normal functions and causing cytoplasmic coagulation, denaturation of enzymes and proteins, and loss of metabolites and ions [43,44]. The efficacy of CEO against the strains tested is of particular interest considering that *E. coli*, which is Gram-negative, has a thick outer membrane layer of lipopolysaccharides covering the cell wall, potentially making it more resistant to hydrophobic substances than Gram-positive bacteria [45,46,47]. Cinnamaldehyde has important anti-adhesive properties against pathogenic *E. coli* [48]. By testing *E. coli* ATCC 25,922 [49], it has been shown that cinnamaldehyde may suppress bacterial growth prolonging the lag phase, may increase cell membrane permeability causing its collapse and leakage of cell contents, and may cause oxidative damage to the bacterial cell membrane. On the other hand, regarding *E. coli* and other Gram-negative bacteria such as *Salmonella* spp., *Pseudomonas* spp., and *Vibrio* spp., cinnamon is able not only to alter ATP-ase activity and thus the permeability of cell membranes, but also to interfere with mitochondrial functions and cell division mechanisms of bacterial cells [50]. In addition, cinnamaldehyde can downregulate genes associated with the flagellar system and biofilm formation [36,51]. In the case of *E. coli* ATCC 8735, exposure to low concentrations of cinnamaldehyde changes its structure and morphology, altering its fatty acid composition and binding directly to genomic DNA [52]. 

The MIC values found in our study ranged from 0.2 to 0.5 µL/mL and 0.3 to 0.8 µL/mL for the strains tested with a cell density of 10^6^ UFC/mL and 10^8^ UFC/mL, respectively, in agreement with a previous study [53]. Variable MIC values were reported in other studies. MICs ranged from 0.8 to 3.2 mg/mL of cinnamaldehyde for *E. coli* strains tested with a cell density of 10^5^ UFC/mL [54], and 1 mg/mL [47] or 2.5 mg/mL [55] for a bacterial cell density of 10^7^ UFC/mL. Finally, 6.25 mg/mL was the minimum concentration of cinnamaldehyde effective in inhibiting the growth of bacteria with a cell density of 10^8^ UFC/mL [56]. The different MIC values found in the experiments could be related to the method used to extract the essential oil, as the amount of cinnamaldehyde in the oil may vary depending on the solvent and the pressure and temperature parameters used in the extraction method [57,58,59]. In addition, the laboratory conditions, such as exposition to high temperatures [60,61], prolonged air contact, and exposure to light, while performing experiments, can cause degradation of cinnamaldehyde and affect its efficacy [61,62,63]. The 100% pure essential oil used in this study was obtained by hydro distillation of cinnamon bark, as it provides a high cinnamaldehyde content (between 52% and 81%), which is mainly responsible for cinnamon antibacterial activity [64,65]. 

In our study, CEO showed efficacy, with no relevant differences in MIC values depending on the serogroup of strains, against APEC strains isolated from layers dead from colibacillosis. Moreover, high antimicrobial efficacy was found by testing the strains at a cell density of 10^8^ UFC/mL, usually found in poultry with colibacillosis [66]. Based on these results, CEO may be useful also for therapeutic purposes other than colibacillosis prevention.

Although there are possible differences depending on the serogroup they belong to [67], APEC strains are more frequently endowed with several genes, associated with pathogenic potential, that encode the bacterium’s resistance to attack by the host’s immune system than non-pathogenic fecal strains [13,68,69]. A close relationship was also found between virulence factors and antibiotic resistance. In fact, genes responsible for antimicrobial resistance are often included in conjugative plasmids that may also carry virulence factor determinants [51]. Considering that antibiotic therapy is one of the main control strategies to reduce morbidity and mortality caused by APEC infections and that frequent use of antibiotics may lead to the selection of resistant strains, cinnamon could be useful in limiting antibiotic use. In addition, natural compounds and cinnamon particularly have different targets in the bacterial cell that require the development of more complex resistance mechanisms [43,70]. Another interesting point seems to be the combination of cinnamon essential oil with common antibiotics to reduce their dosage, preserve their effectiveness, and boost their antibacterial efficacy [71]. Previous studies have shown the synergistic activity of CEO in combination with ampicillin and chloramphenicol against *Staphylococcus aureus* (*S. aureus*)*,* and with chloramphenicol against *E. coli*, resulting in a decrease of MICs of the antibiotics [72]. The synergistic interaction of CEO with piperacillin against *E. coli* [73], and with colistin against multidrug resistant strains of *Pseudomonas aeruginosa* (*P. aeruginosa*) [74] was also found. Similarly, thyme essential oil increases the antibacterial efficacy of piperacillin, cefepime, and meropenem against *P. aeruginosa* compared with the efficacy of the individual drugs [75]. In addition, Italian strawflower *(Helichrysum italicum)* essential oil increases the efficacy of B-lactams, quinolones, and chloramphenicol against *Enterobacter aerogenes*, *E. coli*, and *P. aeruginosa*, reducing their respective MIC values [76]. The combination of tobramycin and tea tree oil and a mixture of fennel essential oil, cefoxitin, mupirocin, co-trimoxazole, and ciprofloxacin showed relevant antibacterial efficacy against ATCC strains of *E. coli* and *S. aureus* [77] and multidrug resistant *S. aureus* strains [78].

In addition, natural plant-derived compounds such as thymol [79] and cinnamaldehyde [80,81], are rapidly excreted through the kidneys and have a short half-life, making animal products safe for human consumption.

Finally, the antimicrobial efficacy of cinnamon could be enhanced by the association with other natural substances, as previously reported for other essential oils. The combination of essential oil of eucalyptus (*Eucalyptus deves*) and coriander (*Coriandrum Sativum*) showed synergy against *Yersinia Enterocolitica* [82]. The combination of three plant-derived phenolic principles, thymol (contained in large quantities in thyme), carvacrol (in thyme and oregano), and eugenol (in cinnamon and cloves) showed greater antimicrobial efficacy against *E. coli*, *Salmonella enteritidis*, *S. aureus,* and *P. aeruginosa* than the individual compounds [83]. Various combinations of oregano, thyme, basil, and marjoram also exhibited an additional efficacy against *Bacillus cereus*, *E. coli*, and *P. aeruginosa* [84]. 

Interestingly, cinnamon is one of the phytogenic feed additives (PFAs) approved by the Food and Drug Administration as an additive in poultry feed [85]. PFAs, in addition to their antimicrobial effect, can stimulate the growth of commensal bacteria in the poultry gut, with beneficial effects on the microbiota [36,86]. Also, the use of CEO as a supplement in the diet of broilers leads to the improvement of intestinal immunocompetence and the increase of villi in the gut mucosal surface [87,88,89]. These effects can indirectly contribute to defending against intestinal infection.

## 5. Conclusions

In conclusion, this study highlighted the antimicrobial efficacy of CEO against *E. coli* belonging to some serogroups which are among the most frequently responsible for avian colibacillosis. Considering that colibacillosis is one of the most recurrent and relevant disease in poultry leading to frequent antibiotic use on farms, cinnamon could be a valid option for preventing the infection, especially when combined with other methods such as the increase of biosecurity measures and the use of vaccines. Based on the results of this study, cinnamon could be also useful for the treatment of avian colibacillosis by minimizing the use of antibiotics. However, further studies are needed to better assess this aspect. In addition, another relevant step, should be the evaluation of the most suitable route of CEO administration to birds under field conditions. 

## Figures and Tables

**Table 1:** Inhibitory effect of cinnamon essential oil against *E. coli* strains (10^8^ CFU/mL) grouped according to the bird species of origin.

Species	N° Strains	Cinnamon Essential Oil (µL/mL)	Strains Inhibited (%)	*p*-Value
Laying hens	91	0.1	0 (0)	<0.001
		0.2	0 (0)	
		0.3	1 (1.09)	
		0.4	59 (64.83)	
		0.5	90 (98.9)	
		0.6	91 (100)	
		0.7	91 (100)	
		0.8	91 (100)	
Turkeys	16	0.1	0 (0)	<0.001
		0.2	0 (0)	
		0.3	2 (12.5)	
		0.4	3 (18.75)	
		0.5	14 (87.5)	
		0.6	16 (100)	
		0.7	16 (100)	
		0.8	16 (100)	
Broilers	10	0.1	0 (0)	<0.001
		0.2	0 (0)	
		0.3	1 (10)	
		0.4	4 (40)	
		0.5	10 (100)	
		0.6	10 (100)	
		0.7	10 (100)	
		0.8	10 (100)	
TOTAL	117	0.1	0 (0)	<0.001
		0.2	0 (0)	
		0.3	4 (3.41)	
		0.4	66 (56.41)	
		0.5	114 (97.43)	
		0.6	117 (100)	
		0.7	117 (100)	
		0.8	117 (100)	

**Table 2:** Inhibitory effect of cinnamon essential oil against *E. coli* strains (10^8^ CFU/mL) grouped according to the serogroup.

Serogroups	N° Strains	Cinnamon Essential Oil (µL/mL)	Strains Inhibited (%)	*p*-Value
O78	54	0.1	0 (0)	<0.001
		0.2	0 (0)	
		0.3	3 (5.56)	
		0.4	27 (50)	
		0.5	53 (98.15)	
		0.6	54 (100)	
		0.7	54 (100)	
		0.8	54 (100)	
O2	37	0.1	0 (0)	<0.001
		0.2	0 (0)	
		0.3	1 (2.7)	
		0.4	26 (70.27)	
		0.5	35 (94.59)	
		0.6	37 (100)	
		0.7	37 (100)	
		0.8	37 (100)	
O139	19	0.1	0 (0)	<0.001
		0.2	0 (0)	
		0.3	0 (0)	
		0.4	7 (36.84)	
		0.5	19 (100)	
		0.6	19 (100)	
		0.7	19 (100)	
		0.8	19 (100)	
O128	7	0.1	0 (0)	<0.001
		0.2	0 (0)	
		0.3	0 (0)	
		0.4	6 (85.71)	
		0.5	7 (100)	
		0.6	7 (100)	
		0.7	7 (100)	
		0.8	7 (100)	

**Table 3:** Inhibitory effect of cinnamon essential oil against *E. coli* strains (10^6^ CFU/mL) grouped according to the bird species of origin.

Species	N° Strains	Cinnamon Essential Oil (µL/mL)	Strains Inhibited (%)	*p*-Value
Laying hens	91	0.1	0 (0)	<0.001
		0.2	9 (9.90)	
		0.3	59 (64.83)	
		0.4	91 (100)	
		0.5	91 (100)	
Turkeys	16	0.1	0 (0)	<0.001
		0.2	1 (6.25)	
		0.3	8 (50)	
		0.4	16 (100)	
		0.5	16 (100)	
Broilers	10	0.1	0 (0)	<0.001
		0.2	1 (10)	
		0.3	5 (50)	
		0.4	10 (100)	
		0.5	10 (100)	
TOTAL	117	0.1	0 (0)	<0.001
		0.2	11 (9.4)	
		0.3	72 (61.53)	
		0.4	117 (100)	
		0.5	117 (100)	

**Table 4:** Inhibitory effect of cinnamon essential oil against *E. coli* strains (10^6^ CFU/mL) grouped according to the serogroup.

Serogroups	N° Strains	Cinnamon Essential Oil (µL/mL)	Strains Inhibited (%)	*p*-Value
O78	54	0.1	0 (0)	<0.001
		0.2	7 (12.96)	
		0.3	47 (87.04)	
		0.4	54 (100)	
		0.5	54 (100)	
O2	37	0.1	0 (0)	<0.001
		0.2	4 (10.81)	
		0.3	25 (67.57)	
		0.4	37 (100)	
		0.5	37 (100)	
O139	19	0.1	0 (0)	<0.001
		0.2	0 (0)	
		0.3	0 (0)	
		0.4	19 (100)	
		0.5	19 (100)	
O128	7	0.1	0 (0)	<0.001
		0.2	0 (0)	
		0.3	0 (0)	
		0.4	7 (100)	
		0.5	7 (100)	

## Data Availability

Data sharing not applicable.
